# Multiple Drilling with Recombinant Human Bone Morphogenetic Protein-2 in Korean Patients with Non-Traumatic Osteonecrosis of the Femoral Head: A Prospective Randomized Pilot Study with a Minimum Two-Year Follow-Up

**DOI:** 10.3390/jcm11195499

**Published:** 2022-09-20

**Authors:** Jun Young Park, Byung Woo Cho, Hyuck Min Kwon, Woo-Suk Lee, Kwan Kyu Park

**Affiliations:** 1 Department of Orthopedic Surgery, Yongin Severance Hospital, Yonsei University College of Medicine, Yongin 16995, Korea; 2 Department of Orthopedic Surgery, Gangnam Severance Hospital, Yonsei University College of Medicine, Seoul 06273, Korea; 3 Department of Orthopedic Surgery, Severance Hospital, Yonsei University College of Medicine, Seoul 03722, Korea

**Keywords:** osteonecrosis of the femoral head, joint-preservation procedure, multiple drilling, recombinant bone morphogenetic protein-2, β-tricalcium phosphate

## Abstract

We sought to determine whether multiple drilling (MD) combined with the injection of recombinant human bone morphogenetic protein-2 (rhBMP-2) and β-tricalcium phosphate (β-TCP) could improve survival of the femoral head in pre-collapse lesions of non-traumatic osteonecrosis of the femoral head (ONFH) as compared with MD alone. We conducted a single-site, off-label, comparative and prospective cohort study between November 2017 and May 2019. We enrolled 25 hips (25 patients) with non-traumatic ONFH (Ficat–Arlet stage 2A or less). We performed a survival analysis, and the primary outcome was the occurrence of femoral head collapse on follow-up radiograph. Our cohort consisted of 11 men and 9 women of age 52.5 ± 8.8 years and a body mass index of 24.3 ± 3.0 kg/m^2^. The femoral heads were preserved in 9 hips (45.0%) and collapsed in 11 hips (55.0%) at the final follow-up; mean survival to collapse was 6.9 (range 2.8–13.5) months. There were no significant differences in the survival of the femoral head between the MD alone group and the MD with rhBMP-2 and β-TCP group (five hips survived, 50% vs. four hips survived, 40%, respectively; *p* = 0.83). MD combined with the injection of rhBMP-2 and β-TCP did not improve femoral head survival compared to MD alone in the pre-collapse non-traumatic ONFH lesion.

## 1. Introduction

Osteonecrosis of the femoral head (ONFH) is a disabling and progressive disease affecting adults younger than 50 years of age [1]. ONFH accounts for approximately 10% of all total hip arthroplasty (THA) surgeries, and there are approximately 10,000 to 20,000 new cases of ONFH annually in the United States [2,3]. The annual prevalence exceeded 10,000 in South Korea and Japan [4,5]. Appropriate early intervention before collapse of the femoral head is crucial for a good prognosis in ONFH [6,7]. Without an effective early intervention, approximately 60% of the femoral heads affected by ONFH become symptomatic or collapse, eventually requiring THA [1,5].

The pathogenesis of ONFH remains unclear; however, imbalance of bone remodeling caused by a vascular disruption, thrombus formation, and multifactorial causes are potential mechanisms of ONFH [8,9,10]. Traumatic events such as displaced femoral neck fractures and hip dislocations compromising the supply of retinacular arteries lead to bone ischemia [11]. Further, in addition to long-term corticosteroid use and excessive alcohol intake, coagulopathies, such as congenital afibrinogenemia and hemophilia, hemoglobinopathies, such as sickle cell anemia and thalassemia, and malignancies including myeloproliferative disorders are associated with ONFH [12,13,14,15,16].

Since its first attempt in 1985, core decompression (CD) has been the most commonly used procedure for early ONFH, with an over 60% survival rate for pre-collapse lesions involving less than 30% of femoral head volume [2,6,17,18,19]. As a less invasive technique to reduce the risk of CD-induced fracture, a multiple drilling (MD) technique using a small diameter of 3–8 mm was developed [20]. To support the mechanical properties of the sub-chondral surface, vascularized and non-vascularized bone grafts have been implanted in the necrotic lesion along with CD [21]. In addition, to improve the ability of growth factors to strengthen bone remodeling, various biotechnological adjuvants, such as adjunctive cell therapy, recombinant human bone morphogenetic proteins (rhBMP), and synthetic bone substitutes, have been implemented with CD or MD [22,23,24]. However, to our knowledge, there is a lack of evidence regarding the clinical efficacy of MD combined with rhBMP-2 and beta-tricalcium phosphate (β-TCP) for non-traumatic ONFH.

To determine whether MD combined with the injection of rhBMP-2 and β-TCP could improve survival of the femoral head in patients with pre-collapse lesions of non-traumatic ONFH as compared with MD alone, we conducted a single-site, off-label, comparative and prospective cohort study.

## 2. Materials and Methods

### 2.1. Study Participants Selection

This study was a prospective, single-site, randomized, off-label trial designed to determine the effects of injectable rhBMP-2 and β-TCP combined with MD, compared to that of MD alone, on hip survival in patients with non-traumatic ONFH. Study participants were recruited between November 2017 and May 2019 from a single institution. Eligible patients (1) had a diagnosis of non-traumatic ONFH (Ficat–Arlet stage 2A or less) by radiographic examination [17,25], (2) had no previous medical or surgical therapy for the disease, (3) did not undergo an elective joint-preservation procedure such as CD, and (4) were aged between 20 and 65 years. Among the exclusion criteria were (1) poor general condition due to an infectious disease or accompanying medical illness, (2) a history of malignancy or uncontrolled cardiovascular, endocrinological, and neurovascular diseases, (3) diagnosis of osteoporosis within 1 year, and (4) pregnancy or lactation. The study participants were randomly assigned to undergo MD with injection of rhBMP-2 and β-TCP, or MD alone, after providing written informed consent. We created a randomization sequence using Stata 9.0 (StataCorp, College Station, TX, USA) statistical software and stratified by center with a 1:1 allocation using random block sizes of 2 and 4. This study was approved by the institutional review board of Severance Hospital, Seoul, Korea (1-2017-0026).

### 2.2. Trial Design

Patients assigned to the MD with rhBMP-2 and β-TCP group underwent MD with injection of 0.5 mg of rhBMP-2 plus 1.5 g of β-TCP beads mixed with biodegradable hydrogel (EXCELOS inject; CG Bio, Seongnam, Korea) into the necrotic lesion of the femoral head; patients assigned to the MD alone group underwent only the MD procedure. Patients could be treated using another selected treatment method in cases of disease progression, if the improvement of the disease was unclear, or if a worsening pattern was clearly observed by investigators. All patients completed questionnaires and underwent a physical examination (range of motion and Patrick test), blood tests (complete blood count and comprehensive metabolic panel), and imaging tests. Hip anteroposterior (AP), frog-leg lateral, and cross-table lateral radiographs and magnetic resonance imaging (MRI) were also performed in the screening phase. We measured the combined necrotic angle on the pre-operative MRI and classified each patient as one of four types according to the method by Ha et al. [26]. Femoral heads were classified into four categories according to the degree of the combined necrotic angle: grade 1 (<200°), grade 2 (200° to 249°), grade 3 (250° to 299°), and grade 4 (≥300°). When patients were enrolled, baseline Harris hip score (HHS) and Western Ontario and McMaster Universities arthritis index (WOMAC) score were obtained [27]. Hip AP, frog-leg lateral, and cross-table lateral radiographs were performed post-operatively at 6, 12, and 24 months to assess for femoral head collapse. HHS and WOMAC scores were also obtained at six-month intervals until the last follow-up.

### 2.3. Trial Intervention

The MD procedure was performed as described by Mont et al. [28]. Under an appropriate anesthetic method, depending on the patient’s condition during general and spinal anesthesia, the patient was placed supine on a fracture table. The affected limb was rotated approximately 15° internally. After an approximately 2 cm lateral skin incision at the level of the greater trochanter, the optimal entrance point for drilling was determined using fluoroscopy. A 3.2 mm diameter Steinman pin was introduced into the necrotic lesion, and the direction of the pin was confirmed under fluoroscopic guidance. The pin was then advanced until the tip was positioned within the subchondral bone, approximately 3 to 5 mm from the articular cartilage. Another one or two additional pins were then inserted in parallel into the necrotic area of the femoral head. A delicate effort was made to avoid penetration of the articular cartilage during pin advancement. For those in the MD with rhBMP-2 and β-TCP group, the injection was performed according to the description by Gao et al. [29]. A total of 2 mL of gel-type mixture, comprising 0.5 mg of rhBMP-2 and 1.5 g of β-TCP, was impacted into the drilling holes using a small trephine with an inner diameter of 1.5 mm. After the injection, the lumen of the trephine was confirmed to be empty.

### 2.4. Study End Points

The primary outcome was the occurrence of a femoral head collapse on follow-up radiographs according to the modified system of Ficat and Arlet classification [17,25]. The secondary outcomes included HHS, WOMAC scores, and the clinical failure rate of the operated hips at the final follow-up. We defined the clinical failure rate as the proportion of hips that required THA or that progressed to Ficat stages 2B, 3, and 4.

### 2.5. Statistical Analyses

We used the Wilcoxon rank-sum test and the Fisher exact test for comparing the means and proportions of the selected baseline characteristics (two-tailed; α = 0.05). We defined the survival time as the time elapsed between the initial enrollment and the primary end point or the end of follow-up. We constructed a Kaplan–Meier survival curve to perform the survival analysis. We performed a log-rank test to compare survival distributions between the two groups. We also performed univariate simple and multiple logistic regression with 95% confidence intervals (CIs) to determine the independent risk factors for femoral head collapse. We considered all enrolled hips to be independent in the statistical analysis. We used R software version 4.1.1 (R Foundation for Statistical Computing, Vienna, Austria) for statistical analyses. We did not perform a sample size calculation because we conducted this trial as a pilot study to elucidate the effectiveness of adjuvant therapy combined with that of the conventional MD.

## 3. Results

**Figure 1:**
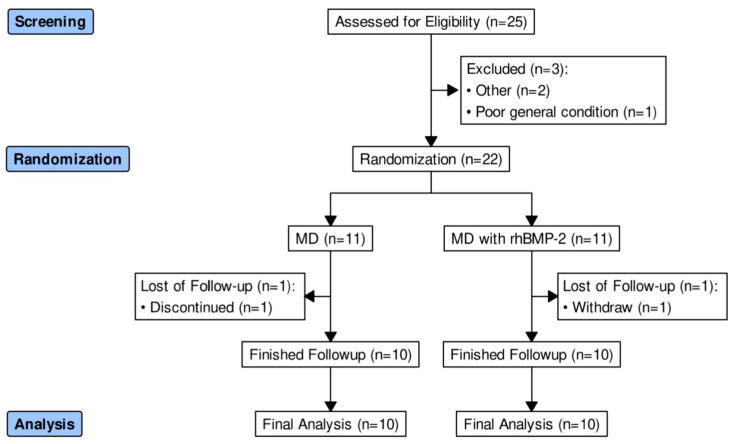
CONSORT diagram. MD, multiple drilling; CONSORT, consolidated standards of reporting trials.

Among 25 hips assessed for eligibility, 3 were excluded according to the exclusion criteria (Figure 1). Two hips were lost to follow-up, one in each of the two groups. Overall, our study enrolled 20 patients (20 hips), comprising 11 men and 9 women of age 52.5 ± 8.8 years and body mass index 24.3 ± 3.0 kg/m^2^. Eleven cases were associated with the risk factor of corticosteroid treatment, four cases with alcohol, and five cases were idiopathic. Fourteen hips were classified as Ficat–Arlet staging grade 2A and six hips as grade 1. Four hips were classified as grade 1 by the Kerboul combined necrotic angle classification, six hips as grade 2, seven hips as grade 3, and three hips as grade 4. The mean follow-up period was 29.5 ± 6.6 months (range 23.3–41.2). Table 1 summarizes the demographic and baseline clinical characteristics of participants in the two groups. Table 2 describes the characteristics of the enrolled participants about clinical failures and radiographic follow-up results.

**Figure 2:**
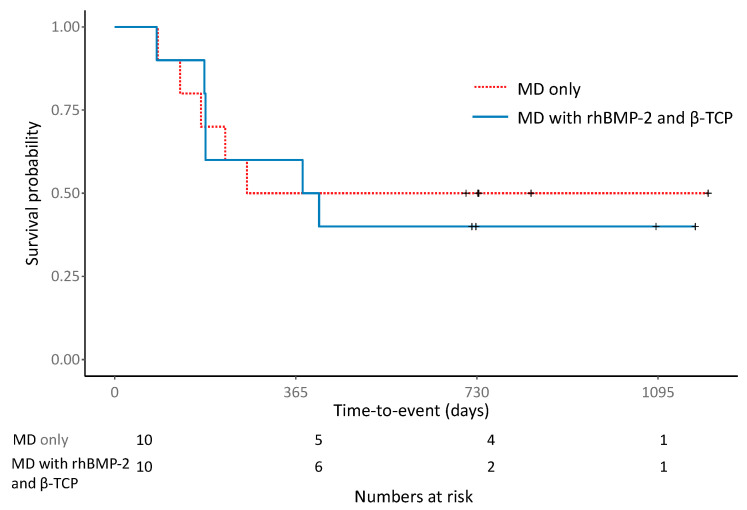
Kaplan–Meier survival curve of femoral head collapse after joint-preservation procedure for pre-collapse ONFH lesion. Red dotted line, MD alone group; blue solid line, MD with rhBMP-2 and β-TCP group. MD, multiple drilling; BMP-2, recombinant human bone morphogenetic protein-2; β-TCP, β-tricalcium phosphate.

The femoral heads were preserved in 9 hips (45.0%) and collapsed in 11 hips (55.0%) at the final follow-up; mean survival duration to collapse was 6.9 (range 2.8–13.5) months (Table 3). There were no significant differences in the survival of the femoral head between the MD alone group and the MD with rhBMP-2 and β-TCP group (five, 50% vs. four, 40%, respectively; *p* = 0.83) (Figure 2). Time to collapse was 5.8 ± 2.3 months in the MD alone group and 7.8 ± 4.2 months in the MD with rhBMP-2 and β-TCP group (*p* = 0.375). Three hips (30.0%) underwent THA at a mean time of 9.9 months in the MD alone group, and three hips (30.0%) underwent THA at a mean time of 13.5 months in the MD with rhBMP-2 and β-TCP group (*p* = 0.428). The mean WOMAC score improved from 45.3 (range 15–72) pre-operatively to 31.5 (range 1–72) at the last follow-up in the MD alone group, and from 39.6 (range 26–70) pre-operatively to 36.8 (range 12–70) at the last follow-up in the MD with rhBMP-2 and β-TCP group; however, there were no significant differences between the two groups in the mean WOMAC scores at the last follow-up (*p* = 0.623). The mean HHS improved from 64.8 (range 48–79) pre-operatively to 71.1 (range 48–90) at the final follow-up in the MD alone group, and from 59.0 (range 39–77) pre-operatively to 61.3 (range 36–87) at the final follow-up in the MD with rhBMP-2 and β-TCP group; however, the differences were not significant (*p* = 0.123) (Table 3).

In the nine hips with clinical success, the respective mean WOMAC scores and HHS significantly improved from 39.6 (range 15–70) and 62.7 (range 1–25) pre-operatively to 10.9 (range 39–77) and 83.4 (range 73–90) post-operatively (*p* = 0.003, *p* < 0.001) (Table 4). In the 11 hips with clinical failure, the mean WOMAC score significantly worsened from 44.8 (range 26–72) pre-operatively to 53.2 (range 39–72) at the final follow-up (*p* < 0.001), and the mean HHS significantly worsened from 61.2 (range 45–79) pre-operatively to 52.7 (range 36–71) at the final follow-up (*p* = 0.001). In addition, the Kerboul combined necrotic angle grades were significantly higher in the 11 hips with a femoral head collapse (*p* = 0.043).

**Figure 3:**
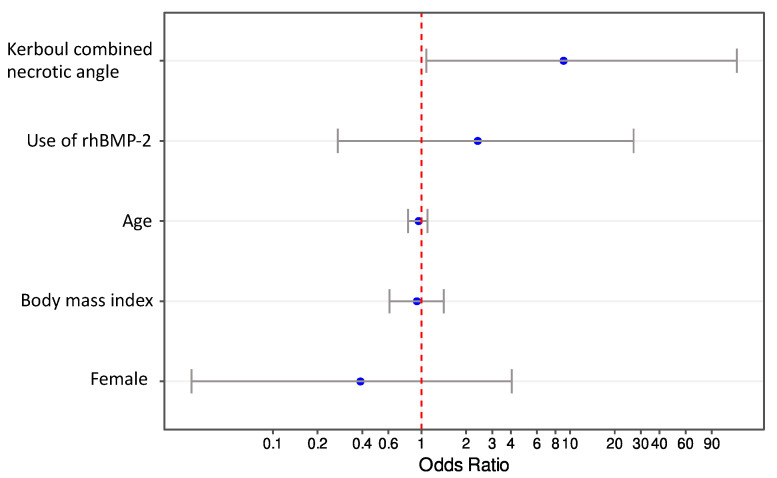
Odds ratio plot for femoral head collapse in non-traumatic osteonecrosis of the femoral head. rhBMP-2, recombinant human bone morphogenetic protein-2.

After accounting for the potentially confounding variables of age, sex, body mass index, Kerboul combined necrotic angle classification, and use of rhBMP-2, the only variable that was independently associated with femoral head collapse in non-traumatic ONFH patients was higher Kerboul combined necrotic angle classification (odds ratio 9.06 (95% CI 0.92–89.6); *p* = 0.042) (Table 5) (Figure 3).

## 4. Discussion

Our study compared the efficacies of MD alone and MD combined with the injection of rhBMP-2 and β-TCP through a minimum two-year follow-up for the treatment of non-traumatic ONFH. Additional use of rhBMP-2 and β-TCP did not improve the survival of the femoral head in the pre-collapse non-traumatic ONFH lesion. The overall survival rate of the joint-preservation procedure in our study was 45% in a median follow-up period of 25.3 months, and the mean survival duration to collapse was 6.9 months.

The survival rate in our study was lower than those previously reported on MD for early ONFH [30,31,32]. Patients with early-stage ONFH tend to be asymptomatic [33]. In this study, because MD was performed on symptomatic patients, the procedural efficacy seemed to have declined. In our subgroup analysis between the femoral head survival and collapse groups, survival seemed independent of the additional use of rhBMP-2 and β-TCP, and measures of the degree of disease progression, such as the size of the necrotic lesion, could be a more significant factor in the prognosis of ONFH. In several reports, the success rate of CD was greatly reduced, and the THA conversion rate was increased in ONFH with large necrotic lesions [34,35]. Because varying rates of success have been reported depending on the study sample size, follow-up period, and type of augmentation [36], the efficacy of MD remains controversial.

**Figure 4:**
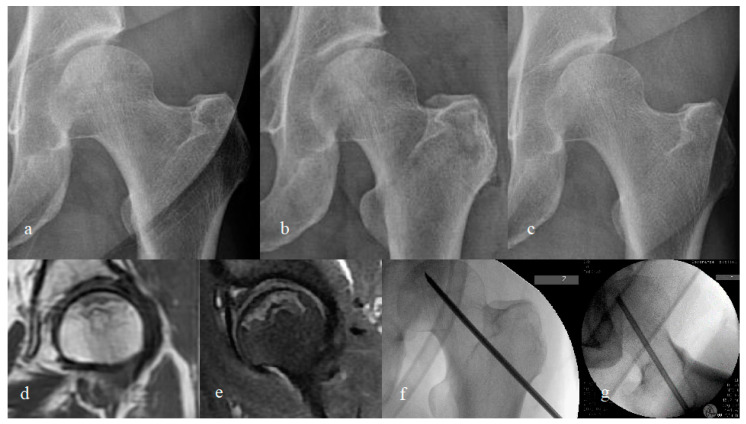
Images of the left hip of a woman who underwent multiple drilling and injections of recombinant human bone morphogenetic protein-2 (rhBMP-2) and β-tricalcium phosphate (β-TCP). (**a**) Pre-operative radiograph (hip anteroposterior view), taken on 3 April 2018; (**b**) immediate post-operative radiograph; (**c**) 3.2 year follow-up radiograph; (**d**) coronal T1-weighted magnetic resonance image; (**e**) sagittal T2-weighted magnetic resonance image; (**f**) intra-operative fluoroscopic image during multiple drilling; (**g**) intra-operative fluoroscopic image of small trephine used for the injection of rhBMP-2 and β-TCP.

However, in the femoral head survival group in our study, the patient-reported outcome measure was significantly increased using MD alone and MD combined with rhBMP-2 and β-TCP (Figure 4). Therefore, there is room to consider the combined use of MD and rhBMP-2 as useful in certain patient groups in the treatment of non-traumatic early ONFH. Several reports have indicated that CD combined with rhBMP-2 improved therapeutic performance compared to conventional CD [6,29,30,37]. Thus, future large-scale, randomized, controlled trials in well-recruited cohorts are needed to elucidate the usefulness of rhBMP-2 as a potential adjuvant.

To optimize the therapeutic outcome of pre-collapse ONFH, many recent studies have combined CD with various adjunctive techniques. Treatment has been reported to increase the survival rate by 78.6% to 90% compared to 48% to 65% with conventional CD alone [6]. In particular, CD using mesenchymal stem cells (MSC) as a biotechnological adjuvant yielded good results in ONFH, and the focus of ONFH treatment has shifted from rhBMP to MSC [38]. However, in some ONFH patients with small necrotic lesions, MD combined with rhBMP could be a good treatment option.

First described by Marshall R. Urist in 1965, BMPs are members of the tumor growth factor-beta superfamily. In the human body, BMP performs important functions in embryonic development and in cellular functions such as growth, differentiation, and apoptosis. In the orthopedic surgery field, BMP has received attention because it controls the proliferation and differentiation of chondrocytes and has a role in osteoinduction, thus promoting bone formation through the differentiation of MSC into osteoblasts [39]. Among several subgroups of BMPs, rhBMP-2 and rhBMP-7 were approved by the Food and Drug Administration and are commercially available for the internal fixation of fractures, spine fusion, and treatment of bone defects. However, complications such as heterotopic ossification, wound complications, inflammatory reactions, and osteoclast ossification have been reported in some cases, especially those involving high doses of rh-BMP [40]. In our study, we observed none of these complications.

Our study has several limitations. First, as a pilot study, a sample size calculation was not performed, and the number of patients in each group was small. Although the validity of the hypothesis has been verified through our study, it deserves further investigation through a follow-up randomized trial with an adequate number of cases to elucidate the validity of rhBMP-2. In addition, our mean follow-up period of 2.5 years was short. However, most femoral head collapses occur within 1 to 2 years, and previous studies have also reported that most clinical failures occurred within 2 years [41]. Therefore, our follow-up period was sufficient to discriminate between the therapeutic effects of the joint-preservation procedures in non-traumatic ONFH treatment.

## 5. Conclusions

In conclusion, MD combined with the injection of rhBMP-2 and β-TCP did not improve femoral head survival in the pre-collapse non-traumatic ONFH lesions. According to the results of samples analyzed, rhBMP-2 requires thoughtful consideration as a potential adjuvant to the conventional MD.

## Figures and Tables

**Table 1:** Demographics of the study cohort.

Characteristics		MD Alone	MD with rhBMP-2 and β-TCP	*p*
Number of hips		10	10	
Age (years)		49.3 ± 8.9	55.6 ± 7.9	0.06
Sex (n, %)	Male	6 (60%)	5 (50%)	1.00
	Female	4 (40%)	5 (50%)	
BMI (kg/m^2^)		24.2 ± 2.2	24.5 ± 3.8	0.91
Side (n, %)	Left	3 (30%)	5 (50%)	0.65
	Right	7 (70%)	5 (50%)	
Ficat–Arlet classification (n, %)	Gr I	3 (30%)	3 (30%)	1.00
	Gr IIA	7 (70%)	7 (70%)	
Associated risk factor (n, %)	Alcohol	1 (10%)	3 (30%)	0.25
	Idiopathic	4 (40%)	1 (10%)	
	Steroid	5 (50%)	6 (60%)	
Kerboul combined necrotic angle (n, %)	Gr 1	3 (30%)	1 (10%)	0.54
	Gr 2	2 (20%)	4 (40%)	
	Gr 3	3 (30%)	4 (40%)	
	Gr 4	2 (20%)	1 (10%)	
Follow-up period (days)		881.1 ± 213.7	916.1 ± 198.6	0.91

MD, multiple drilling; rhBMP-2, recombinant human bone morphogenetic protein-2; β-TCP, β-tricalcium phosphate; BMI, body mass index; Gr, grade.

**Table 2:** Descriptive characteristics of the enrolled participants: clinical failures and radiographic follow-up results.

No.	Age	Sex	BMI	Risk Factor	Direction	Ficat Stage	Kerboul CN Angle	Survival Duration (Days)	Occurrence of FH Collapse
1	59	M	19.8	Idiopathic	Left	2A	1	732	No
2	64	M	29.7	Steroid	Right	2A	2	720	No
3	55	F	26.6	Alcohol	Left	1	3	1196	No
4	53	M	24.4	Alcohol	Left	2A	3	1091	No
5	40	M	24.0	Steroid	Right	2A	4	223	Yes
6	60	F	26.7	Idiopathic	Left	1	1	1170	No
7	59	F	20.4	Steroid	Right	2A	4	183	Yes
8	52	F	26.5	Steroid	Right	1	1	734	No
9	61	F	23.1	Steroid	Right	2A	3	412	Yes
10	52	M	25.7	Idiopathic	Right	2A	2	267	Yes
11	54	M	24.4	Steroid	Left	2A	3	183	Yes
12	32	M	25.6	Steroid	Right	2A	4	132	Yes
13	48	F	24.0	Steroid	Right	2A	3	87	Yes
14	57	F	21.5	Idiopathic	Right	2A	1	708	No
15	49	M	24.8	Alcohol	Left	1	2	379	Yes
16	37	F	19.1	Steroid	Right	1	2	728	No
17	41	M	24.2	Steroid	Right	1	3	174	Yes
18	58	M	30.9	Steroid	Right	2A	3	85	Yes
19	61	F	21.4	Alcohol	Left	2A	2	181	Yes
20	57	M	23.9	Idiopathic	Left	2A	2	839	No

BMI, body mass index; CN angle, combined necrotic angle; FH collapse, femoral head collapse.

**Table 3:** Survival analysis between MD alone and MD with rhBMP-2 and β-TCP groups.

Characteristics		MD Alone	MD with rhBMP-2 and β-TCP	*p*
Femoral head prognosis (n, %)	Preserved	5 (50.0%)	4 (40.0%)	1.00
	Collapsed	5 (50.0%)	6 (60.0%)	
Time to collapse(days)		176.6 ± 71.3	237.2 ± 128.7	0.38
Conversion to THA (n, %)		3 (30.0%)	3 (30.0%)	1.00
WOMAC	PreOp	45.3 ± 19.6	39.6 ± 13.4	0.62
	Last f/u	31.5 ± 29.1	36.8 ± 18.9	0.62
HHS	PreOp	64.8 ± 10.4	59.0 ± 13.5	0.29
	Last f/u	71.7 ± 16.2	61.3 ± 18.2	0.12

MD, multiple drilling; rhBMP-2, recombinant human bone morphogenetic protein-2; β-TCP, β-tricalcium phosphate; THA, total hip arthroplasty; WOMAC, Western Ontario and McMaster Universities arthritis index; HHS, Harris hip score; PreOp, pre-operative; f/u, follow-up.

**Table 4:** Subgroup analysis between femoral head survival and collapse groups.

Characteristics		FH Survival	FH Collapse	*p*
Number of hips		9	11	
Treatment (n, %)	MD alone	5 (55.6%)	5 (45.5%)	1.00
	MD with rhBMP-2 and β-TCP	4 (44.4%)	6 (54.5%)	
Age (years)		54.9 ± 7.6	50.5 ± 9.6	0.36
Sex (n, %)	Male	4 (44.4%)	7 (63.6%)	0.68
	Female	5 (55.6%)	4 (36.4%)	
BMI (kg/m^2^)		24.2 ± 3.5	24.4 ± 2.7	0.91
Side (n, %)	Left	5 (55.6%)	3 (27.3%)	0.41
	Right	4 (44.4%)	8 (72.7%)	
Ficat–Arlet classification (n, %)	Gr I	4 (44.4%)	2 (18.2%)	0.34
	Gr IIA	5 (55.6%)	9 (81.8%)	
Associated risk factor (n, %)	Alcohol	2 (22.3%)	2 (18.2%)	0.14
	Idiopathic	4 (44.4%)	1 (9.1%)	
	Steroid	3 (33.3%)	8 (72.7%)	
Kerboul combined necrotic angle (n, %)	Gr 1	4 (44.4%)	0 (0.0%)	0.04
	Gr 2	3 (33.3%)	3 (27.3%)	
	Gr 3	2 (22.3%)	5 (45.4%)	
	Gr 4	0 (0.0%)	3 (27.3%)	
Time to collapse (days)			209.6 ± 106.4	
WOMAC	PreOp	39.6 ± 17.6 ^a^	44.8 ± 16.1 ^b^	0.62
	Last f/u	10.9 ± 8.3 ^a^	53.2 ± 12.7 ^b^	<0.001
HHS	PreOp	62.7 ± 13.0 ^c^	61.2 ± 11.9 ^d^	0.97
	Last f/u	83.4 ± 4.8 ^c^	52.7 ± 10.1 ^d^	<0.001

^a,d^: *p*-value < 0.01, ^b,c^: *p*-value < 0.001. FH, femoral head; MD, multiple drilling; rhBMP-2, recombinant human bone morphogenetic protein-2; β-TCP, β-tricalcium phosphate; BMI, body mass index; Gr, grade; WOMAC, Western Ontario and McMaster Universities arthritis index; HHS, Harris hip score; PreOp, pre-operative; f/u, follow-up.

**Table 5:** Univariate simple and multiple logistic regression analysis for the predictors of femoral head collapse in non-traumatic osteonecrosis of the femoral head.

Variables	Crude OR (95% CI)	*p*	Adjusted OR (95% CI)	*p*
Use of rhBMP-2				
No	Reference		Reference	
Yes	1.5 (0.26, 8.82)	0.654	2.4 (0.26, 21.79)	0.431
Age	0.94 (0.83, 1.05)	0.268	0.96 (0.83, 1.1)	0.543
Sex				
Male	Reference		Reference	
Female	0.46 (0.08, 2.76)	0.394	0.39 (0.04, 4.15)	0.427
BMI	1.02 (0.76, 1.38)	0.901	0.93 (0.63, 1.39)	0.732
Kerboul combined necrotic angle				
Grade 1 and 2	Reference		Reference	
Grade 3 and 4	9.33 (1.19, 72.99)	0.033	9.06 (0.92, 89.6)	0.042

OR, odds ratio; rhBMP-2, recombinant human bone morphogenetic protein-2; BMI, body mass index.

## Data Availability

Not applicable.

## References

[1] Mont M.A., Zywiel M.G., Marker D.R., McGrath M.S., Delanois R.E. (2010). The natural history of untreated asymptomatic osteonecrosis of the femoral head: A systematic literature review. J. Bone Jt. Surg. Am..

[2] Mont M.A., Salem H.S., Piuzzi N.S., Goodman S.B., Jones L.C. (2020). Nontraumatic osteonecrosis of the femoral head: Where do we stand today?: A 5-year update. J. Bone Jt. Surg. Am..

[3] Moya-Angeler J., Gianakos A.L., Villa J.C., Ni A., Lane J.M. (2015). Current concepts on osteonecrosis of the femoral head. World J. Orthop..

[4] Ikeuchi K., Hasegawa Y., Seki T., Takegami Y., Amano T., Ishiguro N. (2015). Epidemiology of nontraumatic osteonecrosis of the femoral head in Japan. Mod. Rheumatol..

[5] Kang J.S., Moon K.H., Kwon D.G., Shin B.K., Woo M.S. (2013). The natural history of asymptomatic osteonecrosis of the femoral head. Int. Orthop..

[6] Atilla B., Bakırcıoğlu S., Shope A.J., Parvızı J. (2019). Joint-preserving procedures for osteonecrosis of the femoral head. EFORT Open Rev..

[7] Larson E., Jones L.C., Goodman S.B., Koo K.H., Cui Q. (2018). Early-stage osteonecrosis of the femoral head: Where are we and where are we going in year 2018?. Int. Orthop..

[8] Cui Q., Jo W.L., Koo K.H., Cheng E.Y., Drescher W., Goodman S.B., Ha Y.C., Hernigou P., Jones L.C., Kim S.Y. (2021). Arco consensus on the pathogenesis of non-traumatic osteonecrosis of the femoral head. J. Korean Med. Sci..

[9] Petek D., Hannouche D., Suva D. (2019). Osteonecrosis of the femoral head: Pathophysiology and current concepts of treatment. EFORT Open Rev..

[10] Rezus E., Tamba B.I., Badescu M.C., Popescu D., Bratoiu I., Rezus C. (2021). Osteonecrosis of the femoral head in patients with hypercoagulability-from pathophysiology to therapeutic implications. Int. J. Mol. Sci..

[11] Baig S.A., Baig M.N. (2018). Osteonecrosis of the femoral head: Etiology, investigations, and management. Cureus.

[12] Adesina O., Brunson A., Keegan T.H.M., Wun T. (2017). Osteonecrosis of the femoral head in sickle cell disease: Prevalence, comorbidities, and surgical outcomes in california. Blood Adv..

[13] Rodríguez-Merchán E.C. (1996). Effects of hemophilia on articulations of children and adults. Clin. Orthop. Relat. Res..

[14] Simurda T., Kubisz P., Dobrotova M., Necas L., Stasko J. (2016). Perioperative coagulation management in a patient with congenital afibrinogenemia during revision total hip arthroplasty. Semin. Thromb. Hemost..

[15] Thulasidhar A.N., Kumar S., Aroor S., Mundkur S. (2016). Avascular necrosis of femoral head in a child with beta thalassaemia major. J. Clin. Diagn. Res..

[16] Yoon B.H., Jones L.C., Chen C.H., Cheng E.Y., Cui Q., Drescher W., Fukushima W., Gangji V., Goodman S.B., Ha Y.C. (2019). Etiologic classification criteria of arco on femoral head osteonecrosis part 1: Glucocorticoid-associated osteonecrosis. J. Arthroplast..

[17] Ficat R.P. (1985). Idiopathic bone necrosis of the femoral head. Early diagnosis and treatment. J. Bone Jt. Surg. Br..

[18] Hua K.C., Yang X.G., Feng J.T., Wang F., Yang L., Zhang H., Hu Y.C. (2019). The efficacy and safety of core decompression for the treatment of femoral head necrosis: A systematic review and meta-analysis. J. Orthop. Surg. Res..

[19] Roth A., Beckmann J., Bohndorf K., Fischer A., Heiß C., Kenn W., Jäger M., Maus U., Nöth U., Peters K.M. (2016). S3-guideline non-traumatic adult femoral head necrosis. Arch. Orthop. Trauma Surg..

[20] Brown P.J., Mannava S., Seyler T.M., Plate J.F., Van Sikes C., Stitzel J.D., Lang J.E. (2016). Multiple small diameter drillings increase femoral neck stability compared with single large diameter femoral head core decompression technique for avascular necrosis of the femoral head. Surg. Technol. Int..

[21] Sallam A.A., Imam M.A., Salama K.S., Mohamed O.A. (2017). Inverted femoral head graft versus standard core decompression in nontraumatic hip osteonecrosis at minimum 3 years follow-up. Hip Int..

[22] Calori G.M., Mazza E., Colombo A., Mazzola S., Colombo M. (2017). Core decompression and biotechnologies in the treatment of avascular necrosis of the femoral head. EFORT Open Rev..

[23] Kumar P., Shetty V.D., Dhillon M.S. (2020). Efficacy of orthobiologic adjuvants to core decompression for hip preservation in avascular necrosis hip. J. Hip Preserv. Surg..

[24] Zhu S., Zhang X., Chen X., Wang Y., Li S., Qian W. (2021). Comparison of cell therapy and other novel adjunctive therapies combined with core decompression for the treatment of osteonecrosis of the femoral head: A systematic review and meta-analysis of 20 studies. Bone Jt. Res..

[25] Smith S.W., Meyer R.A., Connor P.M., Smith S.E., Hanley E.N. (1996). Interobserver reliability and intraobserver reproducibility of the modified ficat classification system of osteonecrosis of the femoral head. J. Bone Jt. Surg. Am..

[26] Ha Y.C., Jung W.H., Kim J.R., Seong N.H., Kim S.Y., Koo K.H. (2006). Prediction of collapse in femoral head osteonecrosis: A modified kerboul method with use of magnetic resonance images. J. Bone Jt. Surg. Am..

[27] Lane N.E., Hochberg M.C., Nevitt M.C., Simon L.S., Nelson A.E., Doherty M., Henrotin Y., Flechsenhar K. (2015). Oarsi clinical trials recommendations: Design and conduct of clinical trials for hip osteoarthritis. Osteoarthr. Cartil..

[28] Mont M.A., Ragland P.S., Etienne G. (2004). Core decompression of the femoral head for osteonecrosis using percutaneous multiple small-diameter drilling. Clin. Orthop. Relat. Res..

[29] Gao F., Sun W., Guo W., Wang B., Cheng L., Li Z. (2016). Combined with bone marrow-derived cells and rhbmp-2 for osteonecrosis after femoral neck fractures in children and adolescents: A case series. Sci. Rep..

[30] Lieberman J.R., Conduah A., Urist M.R. (2004). Treatment of osteonecrosis of the femoral head with core decompression and human bone morphogenetic protein. Clin. Orthop. Relat. Res..

[31] Mont M.A., Marulanda G.A., Seyler T.M., Plate J.F., Delanois R.E. (2007). Core decompression and nonvascularized bone grafting for the treatment of early stage osteonecrosis of the femoral head. Instr. Course Lect..

[32] Papanagiotou M., Malizos K.N., Vlychou M., Dailiana Z.H. (2014). Autologous (non-vascularised) fibular grafting with recombinant bone morphogenetic protein-7 for the treatment of femoral head osteonecrosis: Preliminary report. Bone Jt. J..

[33] Hsu J.E., Wihbey T., Shah R.P., Garino J.P., Lee G.C. (2011). Prophylactic decompression and bone grafting for small asymptomatic osteonecrotic lesions of the femoral head. Hip Int..

[34] Steinberg M.E., Bands R.E., Parry S., Hoffman E., Chan T., Hartman K.M. (1999). Does lesion size affect the outcome in avascular necrosis?. Clin. Orthop. Relat. Res..

[35] Yoon T.R., Song E.K., Rowe S.M., Park C.H. (2001). Failure after core decompression in osteonecrosis of the femoral head. Int. Orthop..

[36] Liu Z., Yang X., Li Y., Zeng W.N., Zhao E., Zhou Z. (2021). Multiple drilling is not effective in reducing the rate of conversion to total hip arthroplasty in early-stage nontraumatic osteonecrosis of the femoral head: A case-control comparative study with a natural course. BMC Musculoskelet. Disord..

[37] Sun W., Li Z., Gao F., Shi Z., Zhang Q., Guo W. (2014). Recombinant human bone morphogenetic protein-2 in debridement and impacted bone graft for the treatment of femoral head osteonecrosis. PLoS ONE.

[38] Yoon P.W., Kang J.Y., Kim C.H., Lee S.J., Yoo J.J., Kim H.J., Kang S.K., Min J.H., Yoon K.S. (2021). Culture-expanded autologous adipose-derived mesenchymal stem cell treatment for osteonecrosis of the femoral head. Clin. Orthop. Surg..

[39] Ripamonti U., Petit J.C. (2009). Bone morphogenetic proteins, cementogenesis, myoblastic stem cells and the induction of periodontal tissue regeneration. Cytokine Growth Factor Rev..

[40] Shi L., Sun W., Gao F., Cheng L., Li Z. (2017). Heterotopic ossification related to the use of recombinant human bmp-2 in osteonecrosis of femoral head. Medicine.

[41] Wen P., Zhang Y., Hao L., Yue J., Wang J., Wang T., Song W., Guo W., Ma T. (2020). The effect of the necrotic area on the biomechanics of the femoral head—A finite element study. BMC Musculoskelet. Disord..

